# Adult Protective Service's Role in Addressing Older and Dependent Adult Abuse in the Age of COVID

**DOI:** 10.3389/fpubh.2021.659640

**Published:** 2021-06-17

**Authors:** Pi-Ju Liu, Lori Delagrammatikas

**Affiliations:** ^1^School of Nursing and Center on Aging and the Life Course, Purdue University, West Lafayette, IN, United States; ^2^Research-to-Practice Interest Group, National Adult Protective Services Association, Washington, DC, United States; ^3^National Adult Protective Services Association, Washington, DC, United States

**Keywords:** COVID-19 pandemic, adult protective services, elder abuse, abuse of persons with disabilities, neglect, exploitation

## Introduction

In the United States, Adult Protective Services (APS) exist in every state and territory. APS is the only government agency dedicated to addressing older and dependent adult abuse- from the reception and investigation of abuse, assessment of client's service needs, to coordination of healthcare, social, and legal services (1). Most APS programs investigate self-neglect, neglect, physical, emotional (or psychological), sexual, and financial abuse. A smaller number of programs also investigate other types of abuse, such as suspicious death and abandonment (2). All APS programs investigate abuse allegations in the client's home or in a private residence. In 38 states, APS programs also investigate abuse allegations in some types of residential care facilities, such as nursing homes or assisted living facilities. While all APS programs serve older adults age 60 or 65 and above, some programs also serve younger adults with disabilities (3). A range of professionals make up the APS workforce. Although the majority have social work backgrounds, others come from the healthcare and criminal justice sectors [e.g., (4)].

The lack of federal appropriations historically has resulted in variations in state APS programs. APS was developed following the enactment of Title XX of the Social Security Act, now part of the Social Services Block Grant (SSBG). Protective services for children and adults is one of many categories covered by SSBG, but each state has discretion in determining how much funding is to be used in each category (5). APS programs in 37 states opt to use SSBG to finance their APS program to one degree or another. Despite the 2010 enactment of the Elder Justice Act authorizing formula grant funding to states to support their APS programs, no appropriations were provided for this purpose. Apart from the use of SSBG funding for APS, states rely upon state general revenue funds to finance older and dependent adult abuse investigations.

The outbreak of coronavirus disease 2019 (COVID-19) added fuel to the fire of older and dependent adult abuse. Preventive measures, such as self-quarantining, aim to decrease the risk of COVID-19 infection. However, being isolated is a risk factor of abuse (6), creating a catch-22 for this population. A recent study found an 83.6% increase in 1-year abuse prevalence for adults age 60 and older (7). Researchers also voiced concern about abuse against people with disabilities during the pandemic (8). Although 63% of states reported having APS emergency preparedness plans in place before COVID-19, most address challenges brought by natural disasters instead of a pandemic (9). Most older and dependent adult subject to abuse require home-based long-term services and supports, which in itself is a risk factor for COVID-19. Negative physical and psychological health comorbidities as a result of abuse also increase the risk of COVID-19 infection (10, 11). As Han and Mosqueda (12) publicly identified APS as the government agency that protects older adults during the pandemic, this opinion describes APS work during the pandemic, highlighting how the workforce adapted to pandemic related changes to continue protecting older and dependent abuse survivors.

## COVID-19'S Impact on APS in Serving Older and Dependent Abuse Clients

### APS Becomes the Hub for Older and Dependent Adults in Need of Any Services

Many community-based organizations, such as senior centers and home care agencies, have been temporarily closed to prevent the spread of COVID-19. Some service providers, especially those in healthcare, have focused on providing telemedicine to create physical distancing (8, 13). Moreover, collaborating agencies, such as law enforcement and emergency medical services, have been limited in their ability to work with APS due to COVID-19, civil unrest, as well as natural disasters in different parts of the country. With other service providers' limited capacity to intervene, APS often becomes the default agency for the aging and disability systems. When the National Adult Protective Services Association (NAPSA) started hosting weekly forums in March 2020 for state APS administrators to communicate issues and exchange ideas on responses to COVID-19, one of the administrators commented that “When things get tough, everyone leaves it to APS.” In addition to their responsibilities in serving those subject to abuse, neglect, and exploitation, some APS programs started taking on cases involving homelessness and mental illnesses because others were not. Other APS programs received calls from other service providers to provide access to food and healthcare, and to serve as the referral hub to find resources for older and dependent adults.

### Lack of Training and Resources for Personal Protective Equipment (PPE)

A U.S. Department of Homeland Security report (March 28, 2020) (14) categorized public agency workers responding to abuse and neglect of older and dependent adults as first responders who are critically essential personnel. It was recommended that these personnel receive priority in getting PPE, given that they consistently interact with high-risk populations, such as older adults or persons with disabilities who are more likely to have pre-existing conditions or comorbidities. But this categorization was not binding on states; as a result, many APS programs were not given priority in receiving or distributing PPE within their states. APS workers had to put themselves at risk of COVID-19 to conduct investigations and other essential activities for their clients who could not secure help from family or friends. Most APS clients did not have proper PPE– APS reported their clients asking for a facial mask, but APS at that point did not have any for themselves much less to provide to others. As the majority of the APS workforce consists of social workers, training in combating infectious diseases has also been lacking (15). When NAPSA announced a webinar on how to use PPE at the end of March (16), registration exceeded the platform's capacity of 1,000 people within a day. The webinar has since been viewed by over 2,350 people.

### Lack of Consistent Policy

Since APS is not funded by federal dollars dedicated to APS, no federal requirements and policies apply to APS programs. Although the Administration for Community Living (ACL) published the national voluntary consensus guidelines for state APS systems in 2016 (and updated it in 2020) (17), no information on infectious diseases was mentioned. When the pandemic hit, each APS program was on their own. States developed their own COVID-19 policies and the lack of federal guidance ensured inconsistency in policies. For example, the majority of states made changes in their policies regarding face-to-face visits with clients and other parties relevant to a report of abuse, neglect, or exploitation (9), but each state adopted different policies and practices based on statutory requirements. Some states stopped making face-to-face visits; some made face-to-face visits based on supervisor consultation; some continued face-to-face visits for certain types of abuse; while others did not change their practice. Although the flexibility in each state's policy decision allows the response deemed best within each state, the lack of federal guidance made it challenging to determine what would work best or to adopt the best policy in the face of conflicting perspectives of others in the state, resulting in contiguous states having widely different policies. As a result, clients were treated in widely different ways depending upon the state (or even county) in which they live.

### Changes in APS Work

Working to address abuse, neglect, and exploitation, APS staff are aware of worker safety concerns such as unfriendly or even openly hostile perpetrators and witnesses, aggressive animals, infectious pests, and dangerous household hazards. However, the pandemic has highlighted additional safety concerns to the workforce. Workers are worried about being infected, infecting clients, or infecting other staff members and family members as a result of face-to-face investigations (9). In many states, these COVID-19 safety issues are more than concerns, since workers have been infected and cannot work in the field. Many APS programs decided to increase virtual or remote investigations. Challenges in conducting investigations through mobile devices were quickly discovered (18), given that many among this population might not be accustomed to using technology or do not have the technology available. To provide some insight and guidance on how and what to do during virtual investigation, NAPSA (19) interviewed state administrators and provided tips to conduct virtual investigation. Nonetheless, NAPSA stated clearly that virtual investigation is not best practice, and should only be considered when the risk to the worker and client outweighs the benefit of face-to-face investigation. In addition to increased infection rates, other challenges associated with the pandemic include program budget cuts. Since APS operates on state or county funding, including states' discretionary use of SSBG funds, budget cuts have led to involuntarily reduced work time, furloughs, and may lead to reductions in the APS workforce. Many APS programs closed their office buildings to save rent, so workers affected by such decisions will continue to work from home. In addition to the challenges of providing all staff with technology equipment, including a phone or other mobile devices, onboarding new staff is very difficult to do virtually considering the nature of APS investigations.

## Discussion

The COVID-19 pandemic and preventive measures, such as social distancing and stay-at-home orders, have created an isolated living environment. Isolation is a risk factor of abuse, when older or dependent adults abused are often trapped with or only interact with their perpetrators. The occurrence of older and dependent adult abuse has likely increased since the pandemic (7, 8), but many APS programs received fewer reports at the beginning of the pandemic (9). One potential reason for lower numbers of reports is that service providers and mandatory reporters did not see their clients or patients during lockdowns. Although providers have tried to sustain services through phone and other mobile devices, electronic communication, including telemedicine, might not work as well with this population due to limited technology skills, knowledge, or access. In addition, perpetrators may have controlled what was seen or heard by service providers and others, such as neighbors and clergy members who may otherwise interact with an older or dependent adult.

Even though some APS programs have prepared for natural disasters, no APS program (or other service providers for that matter) planned for a pandemic like COVID-19. It is widely recognized that APS workers frequently suffer from vicarious trauma through the exposure to abuse, neglect, and exploitation investigations. The pandemic has exposed workers to additional stressors, given their fears about contracting COVID-19, or infecting their clients or family members. On the practice side, training APS workers to work amidst public health crises, and prioritizing APS workers as essential personnel to receive and distribute PPE would be fundamental in protecting the workforce and clients. Moreover, studies on policy changes and investigation challenges during the pandemic can be helpful in anticipation of another national or global crisis like this pandemic. For example, a study that compares substantiation rates and outcomes before, during, and after the pandemic can inform our understanding of virtual or remote investigation.

In December 2020, the Consolidated Appropriations Act, 2021 (HR 133) appropriated the first-ever federal funding specifically for states' APS programs. From this Act, ACL allocated $93.88 million in funding under the Elder Justice Act, specifically for supporting state APS programs in responding to COVID-19 challenges and related activities. The March 2021 American Rescue Plan Act (HR 1319) provided additional appropriations for the Elder Justice Act with at least $6.12 million going to APS formula grants for Federal Fiscal Year (FFY) 2021, and at least $100 million to be allocated to APS formula grants for FFY 2022. The benefits of this funding will neither be fully achieved nor sustained without congressional commitment to direct, ongoing federal support for states' APS programs. Continuous funding is crucial if APS is to protect our country's older and dependent adults subject to abuse, neglect and exploitation, and to ensure preparation for the next pandemic and other major disasters.

## Author Contributions

P-JL and LD conceptualized the article together. P-JL drafted the article. LD revised and edited the article.

## Conflict of Interest

The authors declare that the research was conducted in the absence of any commercial or financial relationships that could be construed as a potential conflict of interest.
